# Comparison of lateral transperitoneal and retroperitoneal approaches for homolateral laparoscopic adrenalectomy

**DOI:** 10.1186/s12893-021-01422-w

**Published:** 2021-12-20

**Authors:** Zhao Liu, Da-wei Li, Lei Yan, Zhong-Hua Xu, Gang-li Gu

**Affiliations:** Department of Urology, Qilu Hospital, Shandong University, No 107, Wenhuaxi Road, Jinan, 250012 Shandong People’s Republic of China

**Keywords:** Transperitoneal, Retroperitoneal, Laparoscopic, Adrenalectomy

## Abstract

**Background:**

There is a lack of data regarding the appropriateness of transperitoneal and retroperitoneal approaches for homolateral laparoscopic adrenalectomy. The aim of this study is to compare lateral transperitoneal and retroperitoneal approach for left-sided and right-sided laparoscopic adrenalectomy respectively.

**Methods:**

Between January 2014 and December 2019, 242 patients underwent left-sided and 252 patients underwent right-sided laparoscopic adrenalectomy. For left side, transperitoneal approach was used in 132 (103 with tumors < 5 cm and 29 with tumors ≥ 5 cm) and retroperitoneal approach in 110 (102 with tumors < 5 cm and 8 with tumors ≥ 5 cm). For right side, transperitoneal approach was used in 139 (121 with tumors < 5 cm and 18 with tumors ≥ 5 cm) and retroperitoneal approach in 113 (102 with tumors < 5 cm and 11 with tumors ≥ 5 cm). Patient characteristics and perioperative outcomes were recorded. For each side, both approaches were compared for tumors < 5 cm and ≥ 5 cm respectively.

**Results:**

For left-sided tumors < 5 cm, transperitoneal approach demonstrated shorter operative time, less blood loss and longer time to oral intake. For left-sided tumors ≥ 5 cm, the peri-operative data of both approaches was comparable. For right-sided tumors < 5 cm, transperitoneal approach demonstrated shorter operative time and less blood loss. For right-sided tumors ≥ 5 cm, the peri-operative data was comparable.

**Conclusions:**

Lateral transperitoneal and retroperitoneal approach are both effective for laparoscopic adrenalectomy. Lateral transperitoneal approach is faster with less blood loss for tumors < 5 cm.

## Background

Laparoscopic adrenalectomy was first introduced in 1992 by Gagner et al. [1], and has since become a standard treatment for benign adrenal masses. Several minimally invasive approaches have been developed for surgery; among them, lateral transperitoneal and retroperitoneal approaches, are the most frequently performed.

Currently, transperitoneal approach has been accepted as the standard procedure because of its widespread use. Most authors believe that the transperitoneal approach provides a superior view, has more familiar anatomic landmarks, a shorter learning curve, and facilitates resection of larger tumors. Retroperitoneal approach is generally associated with faster recovery of bowel movements and a shorter hospitalization period due to the advantages of preventing mobilization of intra-abdominal organs. However, studies comparing transperitoneal and retroperitoneal approaches have revealed contrasting results. A few studies have associated transperitoneal approach with reduced conversion rate and shorter operative time [2–6]; while other studies have suggested a reduction in blood loss, analgesic use, hospital stay, and overall morbidity with regard to retroperitoneal approach [7, 8]. Rubinstein et al. randomly compared 25 patients using a transperitoneal approach and 32 patients using retroperitoneal approach, and established that the operative time, blood loss, specimen weight, analgesic requirement, hospital stay, and complication rate were similar between the two groups [9]. Another randomized controlled trial compared 21 patients with Cushing’s syndrome using transperitoneal or retroperitoneal approaches and the results revealed no variations in the operative time, analgesic requirement, and hospital stay [10].

The contrasting results could be attributed to small sample sizes, heterogeneity of surgical techniques (e.g. patient position, port placements, and dissection sequence), and the level of experience of the surgeons. Most studies have compared transperitoneal and retroperitoneal approaches bilaterally, although adjacent anatomy of adrenal glands on each side was distinct. Furthermore, tumor size has been demonstrated to be a key factor influencing operative outcomes [11]. To our knowledge, few studies have compared the application of the two approaches in homolateral laparoscopic adrenalectomy based on tumor size. In the present study, we compared lateral transperitoneal and retroperitoneal approaches for homolateral laparoscopic adrenalectomy. The application of both approaches to the left-sided and right-sided laparoscopic adrenalectomy were compared for small (< 5 cm) and large tumors (≥ 5 cm), respectively.

## Methods

### Patient data

The present retrospective and nonrandomized study was approved by the institutional review board for data analysis. Cases with previous history of homolateral abdominal surgery, tumors exhibiting infiltration to surrounding structures on computerized tomography (CT) or metastatic adrenal tumors were excluded from the study. A total of 494 patients were enrolled for the study between January 2014 and December 2019. Among them, 242 and 252 patients underwent left-sided and right-sided laparoscopic adrenalectomy, respectively. In the case of left-sided adrenalectomy, a transperitoneal approach was used to operate 132 patients (103 patients with tumor sizes < 5 cm and 29 patients with tumor sizes ≥ 5 cm) and retroperitoneal approach was used to operate 110 patients (102 patients with tumor sizes < 5 cm and 8 patients with tumor sizes ≥ 5 cm), whereas a transperitoneal approach was used to operate 139 patients (121 patients with tumor sizes < 5 cm and 18 patients with tumor sizes ≥ 5 cm), and a retroperitoneal approach was used to operate 113 patients (102 patients with tumor sizes < 5 cm and 11 patients with tumor sizes ≥ 5 cm) for the right-sided adrenalectomy. The choice of an approach depended on the preference of surgeons. Four surgeons preferred retroperitoneal to transperitoneal approach while the other four surgeons preferred transperitoneal to retroperitoneal approach. All surgeons were experienced in the techniques and had performed more than 100 cases by the year of 2014. The medical records of patients were reviewed and demographic data, histopathological characteristics, operative time, blood loss, intraoperative complications, conversion to open surgery, time to oral intake, and post-operative analgesic use were compared. Pheochromocytoma has been demonstrated to be associated with a longer operative time. Therefore, we recorded the ratio of pheochromocytoma as a histopathological characteristic. Operative time was recorded as skin-to-skin surgical time. A non-steroidal anti-inflammatory drug was used during the postoperative period based on the pain requirements of patients. Parametric data (characterized by normal distribution) were presented as mean ± standard deviation and compared using *t*-test. Non-parametric data were presented as median and min–max range, and comparisons were made using Mann–Whitney U test. Categorical data were compared using χ^2^ test or Fisher’s exact test. Statistical significance was set at *P*-value < 0.05 and all reported *P*-values were two-sided. Data analyses were performed using SPSS 20.0 (SPSS Inc., Chicago, IL, USA).

### Operation methods

#### Lateral transperitoneal laparoscopic adrenalectomy (LTLA)

Patients were placed in semi-lateral decubitus positions with the side of the lesion elevated at 70°. Three trocars were used for the left-sided adrenalectomy and four trocars were used for the right-sided adrenalectomy. A 10-mm supraumbilical horizontal skin incision was made and a Veress needle introduced to insufflate the abdominal cavity to 12 mmHg (Mercury) using CO_2_. A 10-mm trocar was then inserted through a supraumbilical incision to serve as a camera port. Two subcostal ports, 10 and 5 mm, were inserted under direct endoscopic visualization. One of the ports was placed in the midclavicular line and the other in the anterior axillary line. An additional 5-mm trocar was placed below the xiphoid for liver traction during the right-sided laparoscopic adrenalectomy. Sub-hepatic adhesions were divided and the liver was retracted. The posterior parietal peritoneum located above the duodenum was incised longitudinally along the right margin of the vena cava. The incision was then extended outward along the reflection of the posterior peritoneum and liver. An aspirator was gently inserted through the plane between the adrenal gland and vena cava to retract the gland laterally. The arterioles along the medial border were divided using an ultrasonic device. The right adrenal vein was identified, clipped and divided. Subsequently, the remaining attachments along the upper pole of the gland were divided. Finally, the gland was separated from the upper pole of the kidney and resected. In the case of the left-sided procedures, an incision of the spleno-renal and spleno-diaphragmatic ligaments was made deep enough to visualize the fundus of the stomach and the left crus of the diaphragm. The spleno-pancreatic bloc was mobilized medially to expose the adrenal region with the retraction of gravity, and the left renal vein was identified. An aspirator was gently inserted between the adrenal gland and the left renal vein to retract the gland upward. Attachments along the lower border of the gland were gently divided using an ultrasonic device until the left adrenal vein was identified, clipped and divided. Subsequently, the arterioles along the medial border were divided. Finally, the adrenal gland was separated from the upper pole of the left kidney and resected.

#### Lateral retroperitoneal laparoscopic adrenalectomy (LRLA)

Patients were placed in lateral decubitus positions. The table was flexed to expand the operating space between the 12th rib and the iliac crest. A 15.0-mm skin incision was made in the midaxillary line, 1 cm above the iliac crest. The retroperitoneal space was initially created using the index finger of the surgeon and subsequently expanded using a balloon dissector inflated with 700 ml of air. A 10.0-mm trocar was placed in the incision to serve as a camera port. Under direct endoscopic visualization, 12 and 5-mm trocars were inserted below the costal margin in the anterior axillary and the posterior axillary lines, respectively and the Gerota’s fascia was incised. Dissection was initially carried out between the perirenal fat and anterior renal fascia, and then along the psoas muscle until the adrenal gland was identified. The gland was separated from the upper pole of the kidney. The attachment of the upper and medial border of the adrenal gland was divided using an ultrasonic device. The adrenal vein was clipped and transected, and the specimen was retrieved.

## Results

### Left-sided laparoscopic adrenalectomy

#### Cases with tumor sizes < 5 cm

Patients who were subjected to both lateral transperitoneal and retroperitoneal approaches had comparable demographic characteristics (Table 1). Pheocromocytoma accounted for 8.7% and 2.9% of the tumors for transperitoneal and retroperitoneal approaches, respectively (*P* = 0.077). The peri-operative outcomes of both procedures are presented in Table 2. Transperitoneal approach was associated with shorter operative time (90 [35–170] vs 95 [50–220] min, *P* = 0.032) and less blood loss (30 [10–300] vs 60 [20–800] ml, *P* = 0.000). Retroperitoneal approach was associated with shorter time to oral intake (2 [1–5] vs 2 [1–5] days, *P* = 0.000). Intraoperative complications, conversion to open surgery, and use of analgesics between the two approaches were comparable (*P* > 0.05). In addition, bleeding caused by injury to the left renal vein occurred in 1 case of the retroperitoneal group, which required conversion to open surgery. No intraoperative complication and conversion to open surgery occurred in the transperitoneal group.

**Table 1:** Characteristics of patients with tumors < 5 cm on the left side

Group	Age	Sex		BMI (kg/m^2^)	Tumor size (mm)	Tumor type	
		M	F			Pheochromocytoma	Others
LTLA (*n* = 103)	48.8 ± 12.1	45	58	21.1 (17.2–28.0)	20 (3–18)	9	94
LRLA (*n* = 102)	48.8 ± 13.2	38	64	21.5 (17.2–28.2)	17 (5–45)	3	99
*t *(*Z, x*^2^) value	*t* = 0.001	*x*^2^ = 0.881		*Z* = − 0.433	*Z* = − 1.090	*x*^2^ = 3.125	
*P* value	0.999	0.348		0.665	0.276	0.077	

*LTLA* lateral transperitoneal laparoscopic adrenalectomy, *LRLA* lateral retroperitoneal laparoscopic adrenalectomy

**Table 2:** Peri-operative data of patients with tumors < 5 cm on the left side

Groups	Operation time (min)	Blood loss (ml)	Intraoperative complications (n)	Conversion to open surgery (n)	First oral intake (days)	Postoperative analgesic use (n)
LTLA (*n* = 103)	90 (35–170)	30 (10–300)	0	0	2 (1–5)	5
LRLA (*n* = 102)	95 (55–220)	60 (20–800)	1	1	2 (1–5)	3
*t *(*Z,χ*^2^) value	*Z* = − 2.141	*Z* = − 7.504			*Z* = − 4.416	*χ*^2^ = 0.120
*P* value	0.032	0.000	0.498*	0.498*	0.000	0.729

*LTLA* lateral transperitoneal laparoscopic adrenalectomy, *LRLA* lateral retroperitoneal laparoscopic adrenalectomy

*Fisher's Exact Test

#### Cases with tumor sizes ≥ 5 cm

The demographic data of patients who were subjected to both surgical approaches are listed in Table 3. A statistically significant difference (*P* = 0.010) was observed in tumor size (70 [50–110] vs 53 [50–60] mm) between the transperitoneal and retroperitoneal approaches. Pheocromocytoma accounted for 31% and 37.5% of the tumors treated with transperitoneal and retroperitoneal approaches, respectively (*P* = 1.000). The operative time, blood loss, time to oral intake, intraoperative complications, conversion to open surgery, and postoperative use of analgesics between the two approaches were comparable (*P* > 0.05, Table 4). One spleen injury and one descending colon injury occurred in the transperitoneal group; however, both injuries were managed with laparoscopy. One diaphragm injury occurred in the retroperitoneal group, which required conversion to open surgery.

**Table 3:** Characteristics of patients with tumors ≥ 5 cm on the left side

Group	Age (years)	Sex		BMI (kg/m^2^)	Tumor size (mm)	Tumor type	
		M	F			Pheochromocytoma	Others
LTLA (*n* = 29)	41.5 ± 14.3	11	18	23.0 (19.0–30.0)	70 (50–110)	9	20
LRLA (*n* = 8)	41.1 ± 11.1	3	5	22.5 (20.0–27.6)	53 (50–60)	3	5
*t *(*Z, χ*^2^) value	*t* = 0.078			*Z* = − 0.559	*Z* = − 2.584		
*P* value	0.938	1.000*		0.576	0.01	1.000*	

*LTLA* lateral transperitoneal laparoscopic adrenalectomy, *LRLA* lateral retroperitoneal laparoscopic adrenalectomy

*Fisher's exact test

**Table 4:** Peri-operative data of patients with tumors ≥ 5 cm on the left side

Groups	Operation time (min)	Blood loss (ml)	Intraoperative complications (n)	Conversion to open surgery (n)	First oral intake (days)	Postoperative analgesic use (n)
LTLA (*n* = 29)	130 (60–290)	50 (10–600)	2	0	2 (1–4)	1
LRLA (*n* = 8)	115 (80–200)	105 (10–1500)	1	1	2 (1–5)	0
*t *(*Z, χ*^2^) value	*Z* = − 0.351	*Z* = − 1.692			*Z* = − 0.179	
*P* value	0.725	0.091	0.530*	0.216*	0.858	1.000*

*LTLA* lateral transperitoneal laparoscopic adrenalectomy, *LRLA* lateral retroperitoneal laparoscopic adrenalectomy

*Fisher's exact test

### Right-sided laparoscopic adrenalectomy

#### Cases with tumor sizes < 5 cm

The demographic data of patients who underwent both procedures were comparable (Table 5). Pheocromocytoma accounted for 6.2% and 5.9% of the tumors treated with transperitoneal and retroperitoneal approaches, respectively (P = 0.195). Transperitoneal approach was associated with a shorter operative time (70 [25–200] vs 100 [55–210] min, *P* = 0.000) and less blood loss (30 [100–200] vs 50 [5–300] ml, *P* = 0.000; Table 6). Intraoperative complications, conversion to open surgery, time to oral intake, and use of analgesics between the two approaches were comparable (*P* > 0.05). One short hepatic vein injury occurred in the transperitoneal group and one duodenum injury occurred in the retroperitoneal group. However, both injuries were managed with laparoscopy and no conversion to open surgery occurred in both groups.

**Table 5:** Characteristics of patients with tumors < 5 cm on the right side

Group	Age	Sex		BMI (kg/m^2^)	Tumor size (mm)	Tumor type	
		M	F			Pheochromocytoma	Others
LTLA (*n* = 121)	51.8 ± 12.78	42	79	20.53 (16.13–26.32)	20 (5–48)	13	108
LRLA (*n* = 102)	49.47 ± 11.38	45	57	21.27 (16.51–27.19)	20 (7–41)	6	96
*t *(*Z, χ*^2^) value	*t* = 1.426	*χ*^2^ = 2.058		*Z* = − 1.623	*Z* = − 0.196	*χ*^2^ = 1.678	
*P* value	0.155	0.151		0.105	0.845	0.195	

*LTLA* Lateral Transperitoneal Laparoscopic Adrenalectomy, *LRLA* Lateral Retroperitoneal Laparoscopic Adrenalectomy

**Table 6:** Peri-operative data of patients with tumors < 5 cm on the right side

Groups	Operation time (min)	Blood loss (ml)	Intraoperative complications (n)	Conversion to open surgery (n)	First oral intake (days)	Postoperative analgesic use (n)
LTLA (*n* = 121)	70 (25–200)	30 (10–200)	1	0	2 (1–5)	0
LRLA (*n* = 102)	100 (55–210)	50 (5–300)	1	0	2 (1–4)	0
*t *(*Z, χ*^2^) value	*Z* = − 7.251	*Z* = − 6.749			*Z* = − 1.794	
*P* value	0.000	0.000	1.000*		0.073	

*LTLA* Lateral Transperitoneal Laparoscopic Adrenalectomy, *LRLA* Lateral Retroperitoneal Laparoscopic Adrenalectomy

*Fisher's Exact Test

#### Cases with tumor sizes ≥ 5 cm

The demographic data of patients subjected to transperitoneal and retroperitoneal approaches were comparable (Table 7). Pheocromocytoma accounted for 33.3% and 36.4% of the tumors treated with transperitoneal and retroperitoneal approaches, respectively (*P* = 1.000). The operative time, blood loss, intraoperative complications, conversion to open surgery, time to oral intake, and use of analgesics between the two approaches were comparable (*P* > 0.05; Table 8). With reference to transperitoneal approach, extensive adherence between the tumor and the liver was observed in one case with a pheochromocytoma of 110 mm, which led to liver injury and uncontrolled bleeding that required conversion to open surgery. In addition, one colon injury occurred in the transperitoneal group and was managed with laparoscopy. In the retroperitoneal group, one case of severe bleeding caused by liver injury with pheochromocytoma of 53 mm was observed, which required conversion to open surgery.

**Table 7:** Characteristics of patients with tumors ≥ 5 cm on the right side

Group	Age	Sex		BMI (kg/m^2^)	Tumor size (mm)	Tumor type	
		M	F			Pheochromocytoma	Others
LTLA (*n* = 18)	46.17 ± 14.63	9	9	23 ± 2.74	70 (50–110)	6	12
LRLA (*n* = 11)	35.55 ± 11.78	6	5	23.36 ± 2.11	56 (50–110)	4	7
*t *(*Z, χ*^2^) value	*t* = 2.034			*t* = − 0.376	*Z* = − 1.669		
*P* value	0.052	1.000*		0.710	0.095	1.000*	

*LTLA* lateral transperitoneal laparoscopic adrenalectomy, *LRLA* lateral retroperitoneal laparoscopic adrenalectomy

*Fisher's exact test

**Table 8:** Peri-operative data of patients with tumors ≥ 5 cm on the right side

Groups	Operation time (min)	Blood loss (ml)	Intraoperative complications (n)	Conversion to open surgery (n)	First oral intake (days)	Postoperative analgesic use (n)
LTLA (*n* = 18)	146.67 ± 71.72	50 (10–500)	2	1	2 (1–4)	0
LRLA (*n* = 11)	125.45 ± 39.27	60 (20–400)	1	1	2 (1–5)	0
*t *(*Z, χ*^2^) value	*t* = 0.898	*Z* = − 1.287			*Z* = − 0.434	
*P* value	0.377	0.198	1.000*	1.000*	0.664	

*LTLA* lateral transperitoneal laparoscopic adrenalectomy, *LRLA* lateral retroperitoneal laparoscopic adrenalectomy

*Fisher's exact test

## Discussion

The present study revealed that LTLA was superior to LRLA based on operative time and blood loss when applied to small adrenal masses on the right and left sides. The observation could be explained as follows: First, digitally creation of retroperitoneal space seems to be lengthier than establishment of pneumoperitoneum with Veress needle. Second, the transperitoneal approach provided a greater visual field and a larger working space than the retroperitoneal approach. It’s faster to localize adrenal gland using vena cava, upper pole of kidney, pancreas and renal vein as landmarks. By contrast, in the case of LRLA, identification of the adrenal gland is relatively difficult due to its location buried in retroperitoneal fat. Some authors have suggested that mobilization of the spleen and pancreas during left-sided LTLA is time consuming [12]. Nevertheless, we hold a contrasting opinion. The spleen and tail of the pancreas can be easily mobilized in the semi-lateral decubitus position with the traction of gravity, considering that the plane between the pancreas and Gerota’s fascia is loose. Third, LTLA allows for early isolation and division of adrenal vein for both sides. The mobility of the adrenal gland can be greatly enhanced once the adrenal vein has been divided, in turn, facilitating subsequent dissection. Conversely, in LRLA, the kidney often crosses the path of controlling the adrenal vein, and gland mobilization is mandatory prior to reaching the vein. The surgeons usually perform the operation carefully and gently to avoid injuring the adrenal vein before controlling it. Finally, less blood loss could contribute to less operative time and vice versa. We used an aspirator to retract the adrenal gland rather than grasp the gland directly during LTLA, which decreases the likelihood of the gland to tear and could in turn, reduce blood loss. Furthermore, the small veins in retroperitoneal fat could cause extra bleeding in LRLA.

Laparoscopic resection of large tumors is more challenging due to distorted anatomy and high vascular supply. A tumor size of 6 cm and later 8 cm has been considered as an upper limit for laparoscopic adrenalectomy [13]. With increasing operative experience, large tumors are no longer a contraindication, especially when using the transperitoneal approach. Greco et al. [14] reported that the upper limit for adrenalectomy could be as high as 10–14 cm. Several studies have suggested that transperitoneal approach could be performed safely on masses up to 15 cm [15, 16]. An indication of retroperitoneal approach was confined to small and medium-sized adrenal tumors due to the limited working space [17]. As LRLA being wildly accepted and technologically matured in China, its indication has been extended to large tumors[18, 19]. LRLA has even been reported to be safer and faster than LTLA in the treatment of large adrenal tumors [18]. In the present study, we defined tumors with sizes of ≥ 5 cm as “large” tumors as indicated by most authors [18–21]. The maximum tumor size in our study was 110 mm for both approaches. No statistically significant difference was observed in the peri-operative parameters between LTLA and LRLA on the left and right sides for large tumors. Nevertheless, the results should be interpreted with caution because the sample size was relatively small and the tumor sizes of the LTLA group were considerably greater than those of the LRLA group on the left side. Management of large adrenal tumors using retroperitoneal approach remains a controversial subject. Further studies with long-term oncological outcomes should be conducted using large sample sizes.

A few surgeons are of the opinion that LTLA increases the risk of injury to the intra-abdominal viscera [22]. Nevertheless, no statistically significant difference was observed in intraoperative complications between LTLA and LRLA in the present study. In our opinion, transperitoneal approach minimizes injury to the intra-abdominal viscera under direct visualization. However, manipulation of the intra-abdominal viscera, with extensive adhesion in the abdomen using transperitoneal approach should be conducted with caution. Conversely, the identification of intra-abdominal viscera during LRLA is not easily achieved and injuries cannot be completely avoided especially when the local anatomy of large tumors is distorted. In the present study, liver and duodenum injuries occurred during LRLA.

A few studies have also expressed concerns that bowel handling during LTLA could result in a delayed oral intake [1]. A prospective randomized study with a small number of cases revealed that the time to oral intake was considerably shorter in the retroperitoneal group than in the transperitoneal group [23]. In addition, a multicenter study revealed that transperitoneal approach was associated with a considerably longer time to oral intake than the retroperitoneal approach [6]. However, based on our findings, the colon rarely requires mobilization during right-sided LTLA for small tumors. Chiang et al. compared right-sided LTLA and LRLA, and established that LRLA was not superior to LTLA based on time to oral intake [24]. In the present study, only cases with left-sided adrenal tumors of sizes < 5 cm exhibited superiority of LRLA in time to oral intake. Therefore, it is unlikely that LTLA will result in impaired bowel function in right-sided adrenalectomy. Analgesic use, which is a parameter that indicates postoperative pain, is similar between the two approaches. In the present study, the rate of post-operative analgesic use was generally low. Based on our findings, both approaches are associated with minimum or no postoperative pain.

As a retrospective, non-randomized study, this preliminary study had several limitations. First, the results of cases with large tumors could have been compromised due to a small sample size. Second, the present study only compared peri-operative parameters and did not include long-term results such as surgical access site herniation and oncological outcomes. Finally, the selection of operative method depended solely on the preference of the operator. Therefore, prospective randomized studies using large sample sizes are required for more objective results.

## Conclusions

Overall, LTLA and LRLA are effective approaches for adrenalectomy with minimal postoperative pain. LTLA is faster with less blood loss when applied to small tumors while LRLA seems to present comparable peri-operative parameters to LTLA when applied to large tumors. LTLA does not increase the risk of viscera injury and impairment of bowel function in right-sided adrenalectomy.

## Data Availability

The datasets used and analyzed during the current study are available from the corresponding author on reasonable request.
